# The economic crisis in Lebanon and adolescent nutritional health: a multi-mycotoxin exposure and risk assessment of stunting and thinness

**DOI:** 10.3389/fnut.2026.1822160

**Published:** 2026-05-15

**Authors:** Maha Hoteit, Aya Abou Yassin, Rouaa Daou, Nikolaos Tzenios, Joyce Haddad, Bilal Hotayt, André El Khoury

**Affiliations:** 1 PHENOL Research Program, Faculty of Public Health, Section 1, Lebanese University, Beirut, Lebanon; 2 Department of Primary Care and Population Health, University of Nicosia Medical School, Nicosia, Cyprus; 3 INSPECT-LB (Institut National de Santé Publique, d'Épidémiologie Clinique et de Toxicologie-Liban), Beirut, Lebanon; 4 Centre d’Analyses et de Recherche (CAR), Unité de Recherche Technologies et Valorisation Agro-Alimentaire (UR-TVA), Faculty of Sciences, Campus of Sciences and Technologies, Saint-Joseph University of Beirut, Mar Roukos, Lebanon; 5 Faculty of Public Health, Charisma University, London, United Kingdom; 6 Directorate of Preventive Healthcare, Ministry of Public Health, Beirut, Lebanon; 7 Badaro Endoscopic Center, Moarbes Hospital, Beirut, Lebanon

**Keywords:** aflatoxin B1 (AFB1), dietary exposure, Lebanese adolescents, mycotoxins, public health risk

## Abstract

**Introduction:**

The Lebanese diet, rich in cereals, pulses, and dairy, is highly vulnerable to mycotoxin contamination due to the country’s temperate climate and humidity. The ongoing economic crisis in Lebanon has intensified food insecurity and malnutrition, increasing health risks related to mycotoxins.

**Methods:**

A nationally representative cross-sectional study assessed exposure to multiple mycotoxins, including aflatoxin B1 (AFB1), aflatoxin M1 (AFM1), ochratoxin A (OTA), deoxynivalenol (DON), T-2, HT-2, zearalenone (ZEA), and fumonisins (FUM) among 442 Lebanese adolescents (mean age 14.56 years) and related risks, with attention to associations with stunting and thinness. Data were collected via a food frequency questionnaire and 24-h recall, whereas contamination levels were sourced from previous studies.

**Results:**

Results showed a stunting prevalence of 4.07% and a thinness at 2.71%, with adolescents consuming an average of 317.56 g/day of cereals. Contamination analysis revealed that AFM1 in dairy products averaged 0.09 μg/kg, exceeding the European limit, and AFB1 in thyme exceeded Lebanese standards at 11.63 μg/kg. Overall, mytotoxin exposure levels were concerning and were associated with MOE values below 10,000, indicating a major public health risk. Notably, AFB1 exposure was linked to additional estimated liver cancer cases per 100,000 people per year. After adjusting for gender, age category, residence, adolescent education, parents’ education, and monthly income, stunting was significantly associated with the estimated daily intake of AFB1 (*β* = −0.07, *t* = −3.65, *p* < 0.001), OTA (*β* = −0.09, *t* = −4.619, *p* < 0.001), and DON (*β* = −0.001, *t* = −3.373, *p* = 0.001).

**Discussion:**

Thinness was associated with exposure to all mycotoxins; the strongest were observed for DON, OTA, AFM1, and AFB1. These findings underscore the urgent need for control measures and public health policies to mitigate mycotoxin exposure among Lebanese adolescents.

## 1 Introduction

Mycotoxigenic fungi such as *Aspergillus, Alternaria, Fusarium*, *and Penicillium* spp. produce low molecular weight toxic metabolites known as mycotoxins (1), which are highly stable under various conditions (2). These secondary metabolites contaminate agricultural products before, during, and/or after harvest, causing toxicity in animals and humans and imposing an economic strain on the agriculture industry (3). Several environmental factors contribute to fungal invasion and growth, and subsequent mycotoxin production, including temperature, humidity, pH, nutrient availability, and microbial interactions (4), as well as agricultural practices and improper processing and storage techniques (5). Today’s changing climate is also contributing to increased contamination of mycotoxins in food (6). Approximately 400 mycotoxins have been identified so far (7), with aflatoxins, ochratoxins, trichothecenes, zearalenone, fumonisins (FUM), patulin, and citrinin recognized as the most significant (8). Aflatoxins are present in a variety of foods, including cereal grains, spices, oilseeds, tree nuts, milk, and dairy products (9). Ochratoxin A (OTA) contaminates foodstuffs such as cereal grains, dried fruits, chocolate, tea, coffee, beer, and wine (10). Moreover, Tricocthecenes and Zearalenone (ZEA) are frequently found in rice, oats, rye, barley, maize, and wheat (3, 11), whereas FUM are mainly found in maize and its products, and in other grain-based products (12). Mycotoxins exhibit a spectrum of harmful effects, including potential carcinogenic, mutagenic, and teratogenic effects, as well as being nephrotoxic, neurotoxic, cytotoxic, immunosuppressive, and estrogenic (13). According to the International Agency for Research on Cancer (IARC), aflatoxins are classified in Group 1, known to be carcinogenic to humans (14). As the most prevalent aflatoxin among all, aflatoxin B1 (AFB1) is also the most potent genotoxin and carcinogen in nature (15), and its intoxication is recognized as a major non-genetic cause of liver cancer, specifically hepatocellular carcinoma (HCC) (16). Although aflatoxin M1 (AFM1), a hydroxylated metabolite of AFB1, is about 10 times less carcinogenic than AFB1, it can still pose a potential health risk to humans, particularly children, due to their high milk intake, lower body weight, faster metabolism, and underdeveloped excretory organs (17). Furthermore, the IARC classifies OTA as Group 2B “a possible human carcinogen” (18), where its main target organ is the kidney. It was considered a leading factor to Balkan Endemic Nephropathy (BEN) (19), which is characterized by chronic interstitial nephritis commonly associated with urothelial cancer (20). Slowed growth, as well as immunotoxic and hematotoxic effects, are associated with chronic exposure to deoxynivalenol (DON) (21). Furthermore, T-2, which is primarily metabolized into HT-2, is associated with several acute toxic effects, including refusal to eat, stomach necrosis, vomiting, bleeding, and skin inflammation (22). Chronic exposure to T-2/HT-2 increases the risk of infections due to their ability to suppress the immune system (7). For ZEA, chronic exposure leads to reproductive and developmental toxicity, as well as hepatotoxicity and immunotoxicity (11). FUM is linked to severe health issues, including nephrotoxicity, esophageal cancer, hepatocarcinoma, immune system suppression, and neural-tube defects (12). The manifestations of these adverse health effects are influenced by the type of mycotoxin, the dosage and duration of exposure (whether acute or chronic), along with individual properties such as age, gender, body mass index, health status, as well as potential co-occurrence effects upon the simultaneous exposure to other chemicals (23). In response, many countries have implemented legislation and guidelines to control mycotoxin levels in food and feed (24). Despite such efforts, it is estimated that mycotoxins contaminate 60–80% of food crops globally, with around 20% of food grain samples exceeding the European Union (EU) and Codex limits, appearing to confirm the original estimates of 25% of the United Nations Food and Agriculture Organization (FAO) (25). During childhood and adolescence, exposure to mycotoxins has been linked to growth, weight, cognitive, and neurological impairments, developmental delay, and challenges in academic performance (26, 27). Indeed, various studies have shown that chronic dietary exposure to aflatoxins through breast milk, infant formula, and weaning foods in early life is associated with impaired growth in children (15, 28). For example, studies in Benin, Togo, and Gambia showed significant negative associations between aflatoxin exposure and the growth parameters height-for-age Z-score (HAZ), weight-for-age Z-score (WAZ), and weight-for-height Z-score (WHZ) among infants and young children; in which HAZ < -2 identifies stunting, WAZ < -2 identifies underweight, and WHZ < -2 identifies wasting (29, 30). Another study in Tanzania showed a negative association between AFM1 exposure and both HAZ and WAZ among breastfed infants (31). In Kenya, AFB1 was associated with lower WAZ among children (6–12 years) (32). Moreover, exposure to FUM was found to have a negative correlation with HAZ (33). In Lebanon, the temperate climate and high humidity provide optimal conditions for the growth of molds and the production of mycotoxins. Additionally, the Lebanese diet, which relies heavily on cereals and pulses, is highly susceptible to contamination by AFT, OTA, and DON (34). As the risks of chronic mycotoxin exposure to children’s health become more apparent, it is important to assess and predict the potential negative health effects of different mycotoxins (35). However, studies among adolescents, who are at higher risk of exceeding regulatory limits than adults, are scarce. Notably, our research group has previously published a study on malnutrition among Lebanese adolescents showing a stunting prevalence of 6.7% and a thinness prevalence of 4.7% (36), yet no studies have linked these growth impairments to mycotoxin exposure. Therefore, by implementing this study, we aimed to assess the dietary exposure to multiple mycotoxins (AFB1, AFM1, OTA, DON, T-2, HT-2, ZEA, and FUM), to evaluate their health risks, and to determine the association of such exposure with stunting and thinness among Lebanese adolescents during the escalating crisis.

## 2 Materials and methods

### 2.1 Study design and data collection

A cross-sectional survey was conducted in Lebanon between December 2022 and March 2023, encompassing a nationally representative sample of Lebanese school-aged children and adolescents. Participants were selected using a probability cluster sampling method from all Lebanese governorates: Mount Lebanon, Beirut, South Lebanon, North Lebanon, Akkar, Nabatieh, Beqaa, and Baalbek-Hermel. To calculate the required sample size, the single population formula was applied: *n* = [*p* (1 − *p*)] ∗ [(Z∝/2)^2^ /(*e*)^2^], where n is the sample size, p is the probability of adolescents (10–18 years) unable to follow preventive measures of the disease (50%), Z(∝/2) is the reliability coefficient of standard error at a 5% significance level (1.96), and e is the tolerated level of standard error (5%) as described by Hosmer and Lemeshow (37). Based on this formula, a minimum of 400 respondents was needed to ensure sufficient statistical power. Taking a 10% non-response rate into account, we reached a total of 442 Lebanese adolescents.

### 2.2 Inclusion and exclusion criteria

To align with the study’s objectives, participants were selected based on specific eligibility criteria, including being Lebanese, aged 10 to 18 years, and free of chronic diseases. Furthermore, only one adolescent per household was recruited for the study.

### 2.3 Study instruments

A validated 119-item semi-quantitative Food Frequency Questionnaire (FFQ) among adolescents was used in the national consumption survey to assess participants’ dietary intake. This FFQ was previously validated for use among Lebanese adolescents (38), showing good reproducibility and acceptable validity for major food groups. Furthermore, to adhere to international standards and ensure compatibility with global dietary exposure evaluations, food items within each FFQ were classified according to the Global Environment Monitoring System (GEMS/Food) distribution of the World Health Organization (WHO).^1^ The FFQ, supplemented with additional items typically consumed by participants at least once a week, was administered by certified dietitians registered within the Lebanese Order of Dietitians. Moreover, participants completed three 24-h dietary recalls—two on weekdays and one on the weekend. Data was collected through 30-min interviews conducted with adolescents or their caregivers, with mothers typically serving as primary informants for younger participants. The FFQ assessed the frequency of consumption of various foods over the past year, recording data as daily, weekly, and monthly frequencies, and then converted to g/day. To help participants accurately recall their food intake and estimate portion sizes, visual aids and instructions were provided. Through a separate questionnaire, sociodemographic characteristics such as age, residency, and educational level were collected. Anthropometric data, including weight and height, were also collected. Height-for-age Z score (HAZ) and body mass index (BMI)-for-age Z score (BAZ) were calculated using the WHO Anthro-Plus software, developed by the World Health Organization to assess the growth of children and adolescents globally (39). HAZ and BAZ scores <−2 indicate moderate stunting and moderate thinness, respectively. HAZ and BAZ scores <−3 indicate severe stunting and severe thinness, respectively. BAZ score >1 indicates overweight and >2 indicates obesity (40).

### 2.4 Ethical considerations

The study adhered to the ethical guidelines of the Helsinki Declaration. It was approved by the Ethics Committee of Al-Zahraa University Medical Center in Beirut, Lebanon (Reference Nb 10-12-2022).

### 2.5 Mycotoxins occurrence data

Mean contamination level of each food item, its standard deviation (SD), minimum, and maximum values were obtained to assess the dietary exposure to multiple mycotoxins and the related health risks. These data were obtained from the mycotoxins database on occurrence and exposure for the Lebanese food basket, which provides a summary of all studies conducted between 2004 and 2022 in Lebanon (41). Food items were classified into food groups according to the Global Environment Monitoring System (GEMS) distribution. Table 1 represents the mycotoxins examined in food items consumed by the participants.

**Table 1:** Food items and corresponding mycotoxins were analyzed.

Food Items	AFB1	AFM1	OTA	DON	T2/HT-2	ZEA		FUM
Bread	✓	✘	✓	✓	✓	✘		✘
Breakfast cereals	✓	✘	✓	✓	✘	✓		✓
Rice, Keshek, Thyme, Nuts	✓	✘	✓	✘	✘	✘		✘
Pasta, Kaak, Seeds, Olives, Dried fruits	✓	✘	✓	✓	✘	✘		✘
Wheat and Bulgur, Flour	✓	✘	✓	✘	✓	✘		✘
Peas, Beans, Lentils, Chickpeas, Falafel, Corn, Chocolate, Stimulant beverages, Beer	✘	✘	✓	✘	✘	✘		✘
Milk, Yogurt, Labneh, Cheese, Milkshake	✘	✓	✘	✘	✘	✘		✘
Herbs	✓	✘	✓	✓	✓	✓		✘
Cake, Croissant, Doughnut, Alcoholic beverages	✘	✘	✓	✓	✘	✘		✘
Oils	✓	✘	✘	✘	✘	✘		✘

✓, Analyzed; ✘, Not analyzed.

#### 2.5.1 Quality assurance

Studies that primarily used enzyme-linked immunosorbent assay (ELISA), high-performance liquid chromatography (HPLC), ultraperformance liquid chromatography–mass spectrometry (UPLC–MS), and liquid chromatography–mass spectrometry (LC–MS) provided the data for this compilation. In Table 2, the limits of detection (LOD), limits of quantification (LOQ), and recovery rates from each study are summarized as ranges for each mycotoxin in different food groups.

**Table 2:** LOD, LOQ, and recovery rate ranges for each mycotoxin.

Mycotoxins	Food items	LOD (μg/kg)	LOQ (μg/kg)	Recovery Rate
AFB1	Bread	0.0042	0.027	88%
	Breakfast cereals	0.0042–1	0.027	88%
	Rice, Keshek, Thyme, Nuts	0.0042–0.0054	0.018–0.04	82–88%
	Pasta, Kaak, Seeds, Olives, Dried fruits	0.0042–0.01	0.027–0.03	88–96%
	Wheat and Bulgur, Flour	0.0042–0.01	0.027	88–104%
	Herbs	0.0054–0.5	0.018–1.6	60–82%
	Oils	0.01	0.03	96%
AFM1	Milk, Yogurt, Labneh, Cheese, Milkshake	0.001–0.005	0.01–0.03	74–98%
OTA	Bread	0.00012–1	0.00035–0.015	94–97%
	Breakfast cereals	0.0034–1	0.015	94%
	Rice, Keshek, Thyme, Nuts	0.003–0.5	0.014–0.8	90–94%
	Pasta, Kaak, Seeds, Olives, Dried fruits	0.0034–1	0.015–0.21	75–94%
	Wheat and Bulgur, Flour	0.0034–0.12	0.015–0.35	90–104%
	Peas, Beans, Lentils, Chickpeas, Falafel, Corn, Chocolate, Stimulant beverages, Beer	0.05	0.21	75%
	Herbs	0.003–0.5	0.014–1.5	90–93%
	Cake, Croissant, Doughnut, Alcoholic beverages	0.05	0.21	75%
DON	Bread	30	–	–
	Breakfast cereals	18.5–30	–	–
	Pasta, Kaak, Seeds, Olives, Dried fruits	30–62.5	125	85%
	Cake, Croissant, Doughnut, Alcoholic beverages	30–62.5	125	85%
	Herbs	20.5	68.2	98%
T2	Bread	0.39	1.19	105%
	Wheat and Bulgur, Flour	0.39	1.19	105%
	Herbs	0.3	0.8	98%
HT2	Bread	0.83	2.52	100%
	Wheat and Bulgur, Flour	0.83	2.52	100%
	Herbs	0.6	1.9	103%
ZEA	Breakfast cereals	1.75	–	–
	Herbs	0.1	0.3	72%
FUM	Breakfast cereals	25	–	–

### 2.6 Exposure assessment and risk characterization

The Estimated Daily Intake (EDI) was calculated according to the following formula:


$$EDI\;\left(ng/kg\;bw/day\right)=\frac{DI\;\left(kg/d\right)x\;MC\;\left(ng/kg\right)}{BW\;\left(kg\right)}$$

where *DI* is the daily intake of the food item, *MC* is the mean contamination value of the corresponding item, and *BW* is the body weight of the participant. In Supplementary Table S3, EDI for each mycotoxin in each food item was calculated using the formula above using the average weight of all participants (53.66 kg). EDI for each mycotoxin in each food group was also calculated using the same method, with only the mean dietary intake of items having mean contamination data being included in the DI factor. However, in Table 3, EDI for each participant was calculated using their own weight, and the average EDI for early and late adolescents was obtained. This dual approach was deliberately adopted to serve two distinct modeling purposes. The point estimates in Supplementary Table S3 serve as a deterministic screening baseline for evaluating and comparing the relative contribution of isolated food items to total mycotoxin load. Conversely, using individual body weights in Table 3 enables a more refined analysis that prevents the masking of high-risk subgroups among adolescents of varying weights.

**Table 3:** EDI and risk assessment components across different adolescent age groups in Lebanon, by mycotoxin exposure.

	AFB1				AFM1				OTA				
Age categories	Average EDI (ng/kg bw/day)	Total HQ	Total MOE	Total Liver cancer risk (cancer cases/100,000 persons)	Total EDI (ng/kg bw/day)	Total HQ	Total MOE	Total Liver cancer risk (cancer cases/100,000 persons)	Total EDI (ng/kg bw/day)	Total HQ	Total MOE neo	Total MOE non-neo	Total weekly exposure (ng/kg bw)
Early Adolescents (10–13 years)	3.21	0.0391^a^–0.1888^b^	124.60	0.048	0.21	1.05	2702.07	0.003	5.58	0.31	2599.26	847.90	39.05
Late Adolescents (14–18 years)	2.34	0.0285^a^–0.1375^b^	171.08	0.035	0.13	0.67	4280.91	0.0019	3.93	0.22	3685.26	1202.16	27.54
All Adolescents (10–18 years)	2.77	0.0337^a^ − 0.1629^b^	144.19	0.04	0.17	0.86	3313.01	0.012	4.76	0.26	3048.43	994.42	33.30

	DON			T-2			HT-2			ZEA		FUM		
Age categories	Total EDI (ng/kg bw/day)	Total HQ	Total MOE	Total EDI (ng/kg bw/day)	Total HQ	Total MOE	Total EDI (ng/kg bw/day)	Total HQ	Total MOE	Total EDI (ng/kg bw/day)	Total HQ	Total EDI (ng/kg bw/day)	Total HQ	Total MOE
Early Adolescents (10–13 years)	480.58	0.06	436.97	0.14	0.0005	21157.42	0.60	0.0020	4978.22	0.64	0.0026	33.00	0.03	3030.62
Late Adolescents (14–18 years)	315.07	0.04	666.52	0.15	0.0005	19320.76	0.65	0.0022	4546.06	0.24	0.0010	12.47	0.01	8016.27
All Adolescents (10–18 years)	397.83	0.05	527.87	0.15	0.0005	20197.42	0.62	0.0021	4752.33	0.44	0.0018	22.74	0.02	4398.39

^a^Lower Limit of HQ. ᵇUpper Limit of HQ.

For risk characterization, the margin of exposure (MOE), developed by the European Food Safety Authority (EFSA) and the World Health Organization (WHO) (42), was calculated based on the following formula:


$$MOE=\frac{BMDL10\;\left(ng/kg\;bw/day\right)\;}{EDI\;\left(ng/kg\;bw/day\right)}$$

where *BMDL_10_* is the lower confidence limit of the Benchmark Dose, representing the minimum dose that causes cancer at a response of 10%, with a 95% confidence interval. Table 4 represents BMDL_10_ values for each mycotoxin. MOE values ≥ 10,000 pose low health risks, whereas MOE values < 10,000 pose high health risks (43). For OTA, if non-neoplastic MOE is ≥ 200, a low health concern is indicated (44).

**Table 4:** BMDL_10_ and HQ reference values for each mycotoxin (43, 44, 69–74).

Mycotoxin	BMDL_10_ (ng/kg bw/day)	TDI/ARfD (ng/kg bw/day)
AFB1	400	Lower Limit: 17
		Upper Limit: 82
AFM1	570	0.2
OTA (neo)	14,500	18
OTA (non-neo)	4,730	
DON	210,000	8,000
T-2	2,970	300
HT-2	2,970	300
ZEA	–	250
FUM	100,000	1,000

We used the Lower Bound (LB) approach, where non-detects (ND) were set to 0.

Furthermore, the Hazard Quotient (HQ) compares the exposure to a substance with the threshold at which no harmful effects are predicted, such as the accepted daily intake (ADI), tolerable daily intake (TDI), or acute reference dose (ARfD) (42), according to this formula:


$$HQ=\frac{EDI\;\left(ng/kg\;bw/day\right)}{ADI\;or\;TDI\;orARfD\;\left(ng/kg\;bw/day\right)}$$

with reference values for each mycotoxin provided in Table 4. If HQ is > 1, a health risk is indicated, whereas if HQ is < 1, the exposure is unlikely to pose a health risk (45).

Following the guidelines of JECFA (46) for evaluating liver cancer risk from exposure to AFB1 and AFM1, and to estimate the population liver cancer risk attributable to AFB1 exposure, a localized cancer potency factor was calculated instead of the global average. Given that the prevalence of HBsAg in Lebanon is approximately 1.74% among the entire population and 1.55% among those aged less than 20 years old (47), the weighted potency factor was calculated as follows:

1 The original formula used to calculate the Population-Weighted Cancer Potency Factor (P) is a standard method provided by the Joint FAO/WHO Expert Committee on Food Additives (JECFA). It combines the two distinct risk potencies (for people with and without Hepatitis B) based on the prevalence of Hepatitis B in a specific country. The Standard JECFA Formula:

P=(PposxHBsAg)+[(Pnegx(1–H)]

Where:

- P: The final Population-Weighted Potency Factor.
- Ppos: The potency for HBsAg-positive individuals (0.3 cancers per 100,000 per year per ng/kg bw/day).
- Pneg: The potency for HBsAg-negative individuals (0.01 cancers per 100,000 per year per ng/kg bw/day).
- H: The prevalence of Hepatitis B (HBsAg) in the population (expressed as a decimal)

2 Since the real prevalence in Lebanon for adolescents is 1.55% (HBsAg = 0.0155), *p* is approximately equal to 0.015.
3 The calculation of liver cancer risks is based on the following:

3.1 $\begin{array}{l} AFB1\;livercancerrisk=\\ \frac{\;AFB1\;Exposure\;\left(ng/kg\;bw/day\right)\;x\;0.015\;cancercases/100,000\;persons}{1\;\left(ng/kg\;bw/day\right)} \end{array}$

3.2 $\begin{array}{l} AFM1\;livercancerrisk=\\ \frac{AFM1\;Exposure\;\left(ng/kg\;bw/day\right)\;x\;0.015\;cancercases/100,000\;persons}{1\;\left(ng/kg\;bw/day\right)} \end{array}$

Finally, we measured the weekly OTA exposure and compared it with the provisional tolerable weekly intake (PTWI) for OTA of 100 ng/kg bw, as established by JECFA (IARC Publications, n.d.) to assess the risk of kidney diseases.

### 2.7 Statistical analysis

IBM SPSS software, version 26, was used to describe population characteristics with categorical variables presented as frequencies and percentages, and continuous variables as means with standard deviations (SD). For continuous variables, the Independent Samples t-test was used to assess differences in means across genders and age groups, and the One-Way ANOVA test was applied to assess differences in means across variables with more than two groups. For categorical variables, the Chi-Square test and Fisher’s exact test were used to analyze gender differences. To evaluate the relationships between HAZ/BAZ scores and mycotoxin EDI values, Pearson correlation was applied. Before analysis, all mycotoxin EDI variables were log-transformed to address their characteristic right-skewed distribution and to satisfy the normality assumptions required for parametric testing. Simple linear regression models were developed using the stepwise method to examine these relationships, while controlling for potential confounding variables. A significant level of *p* < 0.05 was set for all statistical tests. Additionally, Microsoft Excel 2019 was utilized to convert consumption frequencies (daily, weekly, and monthly) into grams per day per participant. EDI, MOE, HQ, and cancer risk values were calculated for each participant and further analyzed. To account for the potential impact of the probability cluster sampling design on standard errors, geographical location (Governorate) was included as a fixed-effect covariate in all multivariate models. This adjustment was performed to control for within-cluster correlations and to ensure the validity of the reported *p*-values and confidence intervals.

To adjust for sociodemographic confounders, variables that showed a bivariate association with the outcomes at *p* < 0.10 were entered into the stepwise regression models. While we recognize the limitations of automated stepwise selection in the epidemiological literature regarding coefficient bias and inflated Type I errors, this approach was utilized to maintain a parsimonious model given our sample characteristics.

## 3 Results

### 3.1 Participants’ sociodemographic characteristics

Table 5 presents the sociodemographic characteristics of the adolescent population studied, of whom 55.7% were women and 44.3% were men. Most participants were late adolescents (61.1%). Most adolescents were from Mount Lebanon (37.8%), whereas the fewest were from Baalbek-Hermel (5.9%). Education levels varied, with the highest proportion attending secondary school (27.6%). Parents’ education levels were also diverse, with a significant percentage having attended university (31.9%). Furthermore, the mean HAZ ± SD was −0.13 ± 1.15, resulting in an overall stunting prevalence of 4.07%. Additionally, mean BAZ ± SD was 0.38 ± 1.28, indicating a 2.71% overall thinness prevalence.

**Table 5:** Anthropometric and sociodemographic characteristics of the study population.

Characteristics	Overall (*n* = 442, 100%)	Women (*n* = 246, 55.7%)	Men (*n* = 196, 44.3%)	*p*-Value
	Mean ± SD	Mean ± SD	Mean ± SD	
Age	14.56 ± 2.79	15.15 ± 2.78	13.82 ± 2.63	<0.001**
Height	157.56 ± 12.36	156.08 ± 9.74	159.43 ± 14.83	0.007*
Weight	53.66 ± 16.08	52.91 ± 13.49	54.61 ± 18.83	0.289
HAZ	−0.13 ± 1.15	−0.27 ± 1.13	0.05 ± 1.17	0.004*
BAZ	0.38 ± 1.28	0.34 ± 1.11	0.43 ± 1.47	0.507

Age categorization	*N*	%	*n*	%	*n*	%	*p*-value
Age							<0.001**
Early Adolescents (10–13 years old)	172	38.9	77	31.3	95	48.5	
Late Adolescents (14–18 years old)	270	61.1	169	68.7	101	51.5	
Residence							0.004*
Mount Lebanon	167	37.8	82	33.3	85	43.4	
Beirut	25	5.7	19	7.7	6	3.1	
South Lebanon	60	13.6	40	16.3	20	10.2	
North Lebanon	59	13.3	40	16.3	19	9.7	
Akkar	39	8.8	24	9.8	15	7.7	
Nabatieh	35	7.9	15	6.1	20	10.2	
Beqaa	31	7	17	6.9	14	7.1	
Baalbek-Hermel	26	5.9	9	3.7	17	8.7	
Education							<0.001**
Elementary School Level	112	25.3	51	20.7	61	31.1	
Intermediate School Level	119	26.9	52	21.1	67	34.2	
Secondary School Level	122	27.6	74	30.1	48	24.5	
University	89	20.1	69	28	20	10.2	
School address							0.037*
Mount Lebanon	172	38.9	87	35.4	85	43.4	
Beirut	22	5	16	6.5	6	3.1	
South Lebanon	56	12.7	35	14.2	21	10.7	
North Lebanon	59	13.3	40	16.3	19	9.7	
Akkar	39	8.8	24	9.8	15	7.7	
Nabatieh	37	8.4	18	7.3	19	9.7	
Beqaa	31	7	17	6.9	14	7.1	
Baalbek-Hermel	26	5.9	9	3.7	17	8.7	
School type							0.002*
Not Attending School	51	11.5	40	16.3	11	5.6	
Public School	168	38	85	34.6	83	42.3	
Private School	223	50.5	121	49.2	102	52	
Parents education							0.706
Illiterate	19	4.3	9	3.7	10	5.1	
Elementary School Level	54	12.2	31	12.6	23	11.7	
Intermediate School Level	117	26.5	69	28	48	24.5	
Secondary School Level	111	25.1	64	26	47	24	
University	141	31.9	73	29.7	68	34.7	

HAZ classification							0.393
Normal (−2 SD ≤ HAZ ≤ 3 SD)	421	95.2	232	94.3	189	96.4	
Moderate Stunting (−3 SD ≤ HAZ < −2 SD)	13	2.9	10	4.1	3	1.5	
Severe Stunting (HAZ < −3)	5	1.1	2	0.8	3	1.5	
Very Tall (HAZ > 3)	3	0.7	2	0.8	1	0.5	
BAZ classification							0.005*
Normal (−2 SD ≤ BAZ ≤ 1 SD)	295	66.7	175	71.1	120	61.2	
Moderate thinness (−3 SD ≤ BAZ < −2 SD)	10	2.3	4	1.6	6	3.1	
Severe thinness (BAZ < −3 SD)	2	0.5	0	0	2	1	
Overweight (1 SD < BAZ ≤ 2 SD)	82	18.6	49	19.9	33	16.8	
Obesity (2 SD < BAZ ≤ 3SD)	47	10.6	16	6.5	31	15.8	
Severe Obesity (BAZ > 3SD)	6	1.4	2	0.8	4	2	

*Significant at *p*-value < 0.05 for Independent Samples *t*-test; **Significant at *p*-value < 0.001 for Independent Samples *t*-test.

Variables analyzed by the *X*^2^ test. *Significant at *p*-value < 0.05 for *X*^2^ test; **Significant at *p*-value < 0.001 for *X*^2^ test.

Variables analyzed by Fisher’s exact test. *Significant at *p*-value < 0.05 for Fisher’s exact test.

### 3.2 Food consumption patterns across age groups

As represented in Table 6, the most frequently consumed food group among adolescents was cereals and cereal products with a mean consumption of 317.56 ± 158.12 g/day, followed by fruits and fruit products (268.40 ± 197.78 g/day). Conversely, fish and other seafood (6.89 ± 22.04 g/day) were the least frequently consumed group, followed by herbs, spices, and condiments (15.32 ± 12.42 g/day). Among beverages, stimulant and non-alcoholic beverages were consumed in nearly equal quantities, averaging 164.53 ± 170.43 mL/day and 163.27 ± 177.93 mL/day, respectively, whereas alcoholic beverages were the least consumed (2.56 ± 23.60 mL/day). Across age groups, the intake levels for most food groups did not vary significantly. However, late adolescents consumed more vegetables and vegetable products (234.33 ± 147.97 g/day) than early adolescents (206.77 ± 134.31 g/day). They also consumed more herbs, spices, and condiments (16.90 ± 13.00 g/day), meat and meat products (79.42 ± 70.69 g/day), fish and other seafood (8.61 ± 27.59 g/day), and sugar and confectionery (22.99 ± 21.86 g/day) than early adolescents. Late adolescents also consumed more stimulant beverages (186.78 ± 191.42 mL/day) than early adolescents (129.60 ± 123.55 mL/day). Finally, early adolescents reported no consumption of alcoholic beverages, while late adolescents averaged 4.18 ± 30.11 mL/day.

**Table 6:** Dietary intake of food groups by age groups as obtained from the National Consumption Survey.

Mean dietary intake ± SD (g/d)				
Food groups based on GEMS	Overall population *n* = 442	Early adolescents (10–13 years) *n* = 172	Late adolescents (14–18 years) *n* = 270	*p*-value
Cereals and cereal products	317.56 ± 158.12	322.48 ± 153.72	314.42 ± 161.07	0.602
Starchy roots and tubers	66.00 ± 73.33	61.28 ± 74.34	69.02 ± 72.65	0.280
Nuts and oilseeds	44.56 ± 224.78	30.38 ± 58.62	53.59 ± 283.62	0.290
Legumes and pulses	72.27 ± 58.69	70.44 ± 57.57	73.44 ± 59.47	0.602
Vegetables and vegetable products	223.6 ± 143.28	206.77 ± 134.31	234.33 ± 147.97	0.044*
Milk and dairy products	205.07 ± 179.03	223.64 ± 191.56	193.25 ± 169.88	0.082
Fruits and fruit products	268.40 ± 197.78	278.81 ± 203.85	261.76 ± 193.91	0.378
Fruit and vegetable juices	50.77 ± 74.86	49.52 ± 79.53	51.56 ± 71.88	0.780
Herbs, spices, and condiments	15.32 ± 12.42	12.83 ± 11.03	16.90 ± 13.00	<0.001**
Meat and meat products	73.52 ± 66.83	64.27 ± 59.30	79.42 ± 70.69	0.016*
Eggs and egg products	30.07 ± 45.67	31.30 ± 54.71	29.29 ± 38.92	0.653
Fish and other seafood	6.89 ± 22.04	4.19 ± 6.63	8.61 ± 27.59	0.012*
Sugar and confectionery	21.01 ± 19.60	17.91 ± 14.95	22.99 ± 21.86	0.004*
Desserts and snacks	81.29 ± 56.39	79.84 ± 52.03	82.22 ± 59.07	0.665
Fat and oils of animals and vegetables	42.33 ± 22.79	41.12 ± 23.90	43.10 ± 22.06	0.374
Stimulant beverages^a^	164.53 ± 170.43	129.60 ± 123.55	186.78 ± 191.42	<0.001*
Non-alcoholic beverages^a^	163.27 ± 177.93	166.67 ± 193.21	161.10 ± 1567.82	0.749
Alcoholic beverages^a^	2.56 ± 23.60	0.00 ± 0.00	4.18 ± 30.11	0.023*

^a^Beverages are presented in mL/d. ^*^Significant at *p*-value < 0.05 for independent samples *t*-test; **Significant at *p*-value < 0.001 for independent samples *t*-test.

### 3.3 Food contamination data, dietary exposure, and associated health risk assessment by food groups

#### 3.3.1 Cereals and cereal products

The mean AFB1 concentration was 0.46 μg/kg, below the maximum tolerable limit (ML) of 2 μg/kg, set by the European Commission (EC) in cereals and cereal products (41). Breakfast cereals had the highest AFB1 concentration (0.88 μg/kg), whereas pasta had the lowest (0.005 μg/kg). Total AFB1 exposure was 1.9 ng/kg bw/day, with rice contributing the most (0.67 ng/kg bw/ day) and keshek contributing the least (0.00003 ng/kg bw/day). Furthermore, liver cancer cases associated with such exposure were 0.16 cases/100,000 persons/year. MOE values in rice (599.43), wheat and bulgur (1738.87), and flour (3876.33) were below 10,000. For OTA, the mean concentration was 0.85 μg/kg, lower than the ML of 3 μg/kg set by the EC (41), with wheat and bulgur having the highest concentration (1.81 μg/kg) and pasta having the lowest (0.18 μg/kg). A total OTA exposure of 3.68 ng/kg bw/day was reported, with bread contributing the most and keshek the least (0.87 ng/kg bw/day and 0.0004 ng/kg bw/day, respectively). OTA was analyzed in corn; however, it was not detected, indicating it was below the limit of detection and therefore not quantifiable. All MOE values, neo and non-neo, were not calculated. Moreover, the mean DON concentration was 235.21 μg/kg, lower than the ML of 500–750 μg/kg set by EC for cereals and various cereal products (41). Breakfast cereals had the highest DON concentration (632.35 μg/kg), and pasta had the lowest (62.50 μg/kg). DON exposure was highest in bread (245.76 ng/kg bw/day) and lowest from kaak (0.15 ng/kg bw/day), with an average of 467.28 mg/kg bw/day in cereals and cereal products. Additionally, bread and pasta MOE values (854.50 and 5940.44, respectively) were below 10,000. T-2 and HT-2 values in bread, wheat, bulgur, and flour were not detected, indicating that these mycotoxins were not quantified in cereals and cereal products. ZEA and FUM were analyzed in breakfast cereals (48), with concentrations of 15.10 μg/kg and 774.10 μg/kg, respectively. Exposure to ZEA and FUM from breakfast cereals was 0.34 ng/kg bw/day and 17.45 ng/kg bw/day, respectively, and FUM MOE (5732.26) had a value below 10,000.

#### 3.3.2 Nuts and oilseeds

Nuts and oilseeds had an average AFB1 concentration of 0.31 μg/kg, with a higher concentration in nuts (0.40 μg/kg) compared to oilseeds (0.22 μg/kg); all lower than the ML of 2 μg/kg set by EC (41). AFB1 total exposure was 0.07 ng/kg bw/day, with nuts contributing more to this exposure (0.04 ng/kg bw/day) than oilseeds (0.02 ng/kg bw/day). Further, this exposure was associated with 0.01 liver cancer cases/100,000 persons/year. Only the nuts MOE (9968.86) was below 10,000. Average OTA concentration was 0.17 μg/kg. Nuts had a higher concentration (0.25 μg/kg) than oilseeds (0.08 μg/kg). OTA exposure from nuts was 0.03 ng/kg bw/day, compared to 0.01 ng/kg bw/day from oilseeds, with an average total exposure of 0.03 ng/kg bw/day. Neo and non-neo MOE values were above 10,000 and 200, respectively. Moreover, seeds had 62.50 μg/kg DON, with an exposure level of 6.85 ng/kg bw/day and an MOE value above 10,000.

#### 3.3.3 Legumes and pulses

Mean OTA concentration in legumes and pulses was 0.03 μg/kg. Peas, beans, and falafel patty had the lowest concentrations of 0.01 μg/kg, and lentils had the highest (0.105 μg/kg). A total OTA exposure of 0.03 ng/kg bw/day was detected, with the falafel patty contributing the least and lentils the most (0.0007 ng/kg bw/day and 0.0584 ng/kg bw/day). All neo and non-neo MOE values were above 10,000 and 200, respectively.

#### 3.3.4 Milk and dairy products

The average AFM1 concentration in milk and dairy products was 0.09 μg/kg, surpassing the 0.05 μg/kg ML set by the EC for milk. Labneh (strained yogurt) had the highest concentration of 0.2 μg/kg, and milk had the lowest (0.04 μg/kg). Moreover, total AFM1 exposure was 0.18 ng/kg bw/day, with labneh contributing the most (0.06 ng/kg bw/day) and cheese the least (0.02 ng/kg bw/day). This exposure was linked to an additional 0.0015 liver cancer cases/100,000 persons/year and a total MOE level below 10,000 (3206.13).

#### 3.3.5 Fruits and fruit products

Olives and dried fruits were analyzed among fruits and fruit products, with AFB1 concentrations of 0.22 μg/kg for both. Total AFB1 exposure was 0.07 ng/kg bw/day, with olives contributing 0.06 ng/kg bw/day and dried fruits contributing 0.01 ng/kg bw/day. This level of exposure was linked to an additional 0.01 liver cancer cases/100,000 persons/ year. Further, olive MOE (6601.19) was below 10,000. The mean concentration of OTA was 0.08 μg/kg with a total exposure of 0.02 ng/kg bw/day, predominantly from olives. Furthermore, all neo and non-neo MOE values were above 10,000 and 200, respectively. For DON, the mean concentration was 62.5 μg/kg, where olives and dried fruits had the same concentration. Moreover, total exposure was 19.08 ng/kg bw/day, with olives contributing the most (17.21 ng/kg bw/day). All MOE values exceeded 10,000.

#### 3.3.6 Herbs, spices, and condiments

Thyme had a mean AFB1 concentration of 11.63 μg/kg, far above the ML of 2 μg/kg set by the Lebanese Standards Institution (LIBNOR) for thyme and thyme mixes (49), whereas herbs (mint and parsley) had a higher concentration of 18.19 μg/kg, with an overall average of 14.91 μg/kg. A total exposure of 1.37 ng/kg bw/day was reported, with higher exposure from thyme (0.70 ng/kg bw/day) compared to mint and parsley (0.58 ng/kg bw/day). AFB1 exposure was associated with 0.11 liver cancer cases/100,000 persons/year. Additionally, MOE values of thyme (571.95) and mint and parsley (688.52) were below 10,000. Mean OTA concentration was 2.87 μg/kg, with mint and parsley having a higher concentration at 4.41 μg/kg, whereas thyme had a lower concentration of 1.34 μg/kg, below the LIBNOR maximum limit of 3 μg/kg for thyme and thyme mixes. OTA exposure was 0.26 ng/kg bw/day, with mint and parsley (0.14 ng/kg bw/day) contributing more than thyme (0.08 ng/kg bw/day). Furthermore, all neo and non-neo MOE values were above 10,000 and 200, respectively. T-2 and HT-2 were only analyzed in herbs (mint and parsley). T-2 had a mean concentration of 4.40 μg/kg, an exposure of 0.14 ng/kg bw/day, and an MOE of 21128.78, above 10,000. For HT-2, the mean concentration was 18.70, with an exposure of 0.6 ng/kg bw/day and an MOE of 4971.48 ng/kg bw/day, which is below 10,000. Finally, DON and ZEA were analyzed but not detected in herbs, indicating that these mycotoxins were below the limit of detection and therefore not quantifiable. All MOE values, neo and non-neo, were not calculated. 3.3.7. Desserts and Snacks.

The concentration of OTA varied between 0.03 μg/kg in chocolate and 0.51 μg/kg in croissants, with an overall mean of 0.33 μg/kg. Furthermore, OTA total exposure was 0.35 ng/kg bw/day, with the lowest exposure from chocolate (0.01 ng/kg bw/day) and the highest from cake (0.12 ng/kg bw/day). All neo and non-neo MOE values were above 10,000 and 200, respectively. An average DON concentration of 56.67 μg/kg was reported, with an exposure of 28.45 ng/kg bw/day. Doughnuts contributed the least to this exposure, whereas cake contributed the most (4.27 ng/kg bw/day and 16.43 ng/kg bw/day, respectively). All MOE values of the items exceeded 10,000; however, the total MOE value (7381.61) was below 10,000.

#### 3.3.7 Stimulants, non-alcoholic and alcoholic beverages

Stimulant beverages had a mean OTA concentration of 0.51 μg/kg, significantly lower than the ML of 10 μg/kg in soluble coffee, set by the EC (41). Total OTA exposure was 0.52 ng/kg bw/day with both neo and non-neo MOE values exceeding 10,000 and 200, respectively.

In non-alcoholic beverages, AFM1 was detected in milkshake in a concentration of 0.08 μg/kg, above the ML of 0.05 μg/kg for milk, set by the EC (41). Its estimated daily intake was 0.001 ng/kg bw/day, associated with 0.00001 additional live cancer cases/100,000 persons/year and an MOE of 586636.15, exceeding 10,000.

For alcoholic beverages, the OTA level was 0.87 μg/kg. Wine and liquor had a higher OTA level (1.47 μg/kg), yet below the EC ML of wine (2 μg/kg) (41), compared to beer (0.28 μg/kg). Total exposure was 0.03 ng/kg bw/day, and both neo and non-neo MOE values exceeded 10,000 and 200, respectively. Furthermore, the wine and liquor DON level was 52.08 μg/kg, with an exposure level of 0.45 ng/kg bw/day and an MOE value (463030.19) exceeding 10,000.

#### 3.3.8 Fat and oils of animals and vegetables

No detectable levels of AFB1 in oils were reported (21). This mycotoxin was below the limit of detection and therefore not quantifiable. All MOE values, neo and non-neo, were not calculated.

#### 3.3.9 HQ and weekly OTA exposure assessment in all food groups

All food groups recorded HQ values below 1, and weekly OTA exposure levels below the PTWI of 100 ng/kg bw established by JECFA (IARC Publications, n.d.).

### 3.4 Total multi-mycotoxin exposure and associated risks

#### 3.4.1 AFB1

Among all food groups, total AFB1 exposure was 3.41 ng/kg bw/day, as shown in Table 7. Cereals and cereal products contributed the most to this exposure (1.90 ng/kg bw/day), followed by herbs, spices, and condiments (1.37 ng/kg bw/day). Conversely, fruit and fruit products, as well as nuts and oilseeds, contributed the least, with only 0.07 ng/kg bw/day each. Moreover, total AFB1 exposure was linked to an additional 0.28 liver cancer cases/100,000 persons/year and an alarming MOE value of 117.47, significantly lower than 10,000. However, all HQ values were below 1.

**Table 7:** Co-occurrence of mycotoxins in the Lebanese food basket.

	AFB1				AFM1				OTA					
Food Groups	Total EDI (ng/kg bw/day)	Total HQ	Total MOE	Total Liver cancer risk (cancer cases/100,000 persons)	Total EDI (ng/kg bw/day)	Total HQ	Total MOE	Total Liver cancer risk (cancer cases/100,000 persons)	Total EDI (ng/kg bw/day)	Total HQ	Total MOE neo	Total MOE non-neo	Total Weekly exposure (ng/kg bw)	Total MOE
Cereal and cereal-based products	1.90	0.02^a^–0.11^b^	210.50	0.028	–	–	–	–	3.68	0.20	3937.57	1284.46	25.78	5732.26
Nuts and oilseeds	0.07	0.0008^a^–0.0038^b^	6146.67	0.0010	–	–	–	–	0.03	0.0019	418625.81	136558.63	0.24	–
Legumes and pulses	–	–	–	–	–	–	–	–	0.03	0.0019	434946.33	141882.49	0.23	–
Milk and dairy products	–	–	–	–	0.18	0.89	3206.13	0.0027	–	–	–	–	–	–
Fruits and fruit products	0.07	0.0008^a^–0.004^b^	5955.46	0.001	–	–	–	–	0.02	0.0014	593684.60	193664.01	0.17	–
Herbs, spices, and condiments	1.37	0.02^a^–0.08^b^	291.40	0.02	–	–	–	–	0.26	0.01	54819.63	17882.54	1.85	–
Desserts and snacks	–	–	–	–	–	–	–	–	0.35	0.02	41789.46	13632.01	2.43	–
Stimulant beverages	–	–	–	–	–	–	–	–	0.52	0.03	27817.79	9074.36	3.65	–
Non-alcoholic beverages	–	–	–	–	0.0010	0.0049	586636.15	0.000015	–	–	–	–	–	–
Alcoholic beverages	–	–	–	–	–	–	–	–	0.03	0.0019	426965.73	139279.17	0.24	–
Total	3.41	0.04^a^–0.2^b^	117.47	0.05	0.18	0.89	3188.71	0.0027	4.94	0.27	2934.30	957.19	34.59	5732.26

	DON			T-2			HT-2			ZEA		FUM	
Food groups	Total EDI (ng/kg bw/day)	Total HQ	Total MOE	Total EDI (ng/kg bw/day)	Total HQ	Total MOE	Total EDI (ng/kg bw/day)	Total HQ	Total MOE	Total EDI (ng/kg bw/day)	Total HQ	Total EDI (ng/kg bw/day)	Total HQ
Cereal and cereal-based products	467.28	0.06	449.41	0.00	0.00	0.00	0.00	0.00	0.00	0.34	0.0014	17.45	0.02
Nuts and oilseeds	6.85	0.0009	30654.24	–	–	–	–	–	–	–	–	–	–
Legumes and Pulses	–	–	–	–	–	–	–	–	–	–	–	–	–
Milk and dairy products	–	–	–	–	–	–	–	–	–	–	–	–	–
Fruits and fruit products	19.08	0.0024	11005.68	–	–	–	–	–	–	–	–	–	–
Herbs, spices, and condiments	0.00	0.00	0.00	0.14	0.0005	21128.78	0.60	0.0020	4971.48	0.00	0.00	-	-
Desserts and snacks	28.45	0.0036	7381.61	–	–	–	–	–	–	–	–	–	–
Stimulant beverages	–	–	–	–	–	–	–	–	–	–	–	–	–
Non-alcoholic beverages	–	–	–	–	–	–	–	–	–	–	–	–	–
Alcoholic beverages	0.45	0.0001	463030.19	–	–	–	–	–	–	–	–	–	–
Total	522.11	0.07	402.21	0.14	0.0005	21128.78	0.60	0.0020	4971.48	0.34	0.0014	17.45	0.02

^a^Lower Limit of HQ. ᵇUpper Limit of HQ.

#### 3.4.2 AFM1

Total AFM1 exposure was 0.18 ng/kg bw/day, associated with 0.0015 liver cancer cases/100,000 persons/year. Milk and dairy products were the major contributors, whereas milkshakes, as non-alcoholic beverages, contributed only 0.001 ng/kg bw/day to this exposure. Furthermore, the total MOE was below 10,000 at 3188.71, and HQ was below 1.

#### 3.4.3 Ota

OTA exposure totaled 4.94 ng/kg bw/day, with cereals and cereal products being the main contributors (3.68 ng/kg bw/day), whereas fruits and fruit products contributed the least (0.02 ng/kg bw/day). Additionally, total weekly OTA exposure was 34.59 ng/kg bw, below the PTWI of 100 ng/kg bw, and all HQ values were below 1. Non-neo MOE had a value of 957.19, above 200, indicating no risk. However, neo MOE, which was mainly influenced by the consumption of cereals and cereal products, was concerningly low (2934.30).

#### 3.4.4 Don

Total DON exposure was 522.11 ng/kg bw/day, with cereals and cereal products being the main contributing group. This exposure was associated with a low MOE value at 402.21, indicating a major risk, particularly from cereals and cereal products (449.41) and desserts and snacks (7381.61). All HQ values were below 1.

#### 3.4.5 T-2 and HT-2

Total T-2 and HT-2 exposures were 0.14 ng/kg bw/day and 0.60 ng/kg bw/day, respectively, with herbs, spices, and condiments being the only contributing group. HQ values were all below 1. Furthermore, the total T-2 MOE value was 21128.78, above 10,000, indicating no risk. However, the HT-2 MOE value was alarming, at 4971.48.

#### 3.4.6 ZEA and FUM

ZEA and FUM total exposures were 0.34 ng/kg bw/day and 17.45 ng/kg bw/day, respectively, with cereals and cereal products the only contributing group. HQ values were below 1. Moreover, FUM MOE level was below 10,00 with a value of 5732.26, indicating a potential risk.

### 3.5 Total exposure and risk assessment by age groups

As represented in Table 3, the average EDI of AFB1, AFM1, OTA, DON, ZEA and FUM were higher in the early adolescents group compared to the late adolescents group, with early adolescents having an average EDI of 3.21 ng/kg bw/day for AFB1 compared to 2.34 ng/kg bw/day in late adolescents, 0.21 ng/kg bw/day of AFM1 compared to 0.13 ng/kg bw/day, 5.58 ng/kg bw/day of OTA compared to 3.93 ng/kg bw/day, 480.58 ng/kg bw/day of DON compared to 315.07 ng/kg bw/day, 0.64 ng/kg bw/day of ZEA compared to 0.24 ng/kg bw/day, and 33 ng/kg bw/day of FUM compared to 12.47 ng/kg bw/day. However, EDI of T-2 and HT-2 were slightly lower in the early adolescents group compared to the late adolescents’ group, with T-2 at 0.14 ng/kg bw/day in early adolescents compared to 0.15 ng/kg bw/day in late adolescents, and HT-2 at 60 ng/kg bw/day in early adolescents compared to 65 ng/kg bw/day in late adolescents. This suggests that early adolescents are at higher risk of exposure to mycotoxins compared to late adolescents. Furthermore, all HQ values were below 1 in both age groups. Except for T-2, all MOE values were below 10,000 in both age groups, indicating a major health risk. Lastly, AFB1 exposure was associated with higher liver cancer cases compared to AFM1, especially among early adolescents (0.05 liver cancer cases/100,000 persons/year).

### 3.6 Factors associated with HAZ and BAZ scores among adolescents

Table 8 presents the Pearson correlation coefficients (r) examining the linear relationships between the standardized growth Z-scores and the log-transformed estimated daily intakes of mycotoxins.

**Table 8:** Correlation analysis of HAZ and BAZ scores with mycotoxins’ EDI.

Mycotoxin EDI	HAZ		BAZ	
	Pearson Correlation	Sig. (2-tailed)	Pearson Correlation	Sig. (2-tailed)
AFB1 EDI	−0.150**	0.002	−0.208**	<0.001
AFM1 EDI	0.012	0.801	−0.272**	<0.001
OTA EDI	−0.176**	0.000	−0.290**	<0.001
DON EDI	−0.102*	0.032	−0.307**	<0.001
T-2 EDI	−0.098*	0.040	−0.101*	0.034
HT-2 EDI	−0.098*	0.040	−0.101*	0.034
ZEA EDI	−0.057	0.235	−0.105*	0.027
FUM EDI	−0.057	0.235	−0.105*	0.027

^*^Correlation significant at *p* < 0.05; ^**^Correlation significant at *p* < 0.01. It presents the Pearson correlation coefficients ($r$) examining the linear relationships between the standardized growth Z-scores and the log-transformed estimated daily intakes of mycotoxins.

As Table 8 shows, HAZ scores had a significant negative correlation with AFB1 EDI (*p* = 0.002), OTA EDI (*p* < 0.001), DON EDI (*p* = 0.032), T-2 EDI (*p* = 0.04), and HT-2 EDI (*p* = 0.04). Moreover, the analysis revealed a significant difference in HAZ scores between genders (*p* = 0.004) and age groups (*p* = 0.000). Additionally, significant differences in HAZ scores were associated with variations in residence (*p* = 0.027), adolescents’ education (*p* = 0.003), parents’ education (*p* = 0.022), and monthly income (*p* < 0.001) (Supplementary Table S1).

As for BAZ score, it showed a negative correlation with AFB1 EDI (*p* < 0.001), AFM1 EDI (*p* < 0.001), OTA EDI (*p* < 0.001), DON EDI (*p* < 0.001), T-2 EDI (*p* = 0.034), HT-2 EDI (*p* = 0.034), ZEA EDI (*p* = 0.027) and FUM EDI (*p* = 0.027). No sociodemographic factors were associated with BAZ (Supplementary Table S1).

### 3.7 Determinants of adolescents’ stunting and thinness: simple linear regression analysis

As shown in Table 9, unadjusted models were applied to both HAZ and BAZ scores with each mycotoxin as the independent variable. Adjusted models, with HAZ as the outcome, included individual mycotoxin, gender, age category, residence, adolescent education, parents’ education, and monthly income. However, no adjusted models with BAZ as the outcome were applied since none of the sociodemographic variables were significantly associated with BAZ. Adjusted models performed better than unadjusted ones, as evidenced by the higher *R*^2^ value (Supplementary Table S2).

**Table 9:** Linear regression analysis of HAZ and BAZ scores with mycotoxins’ EDI.

			HAZ						BAZ					
			Unstandardized coefficients		Standardized coefficients B	*t*	*p*-value	VIF	Unstandardized coefficients		Standardized coefficients B	*t*	*p*-value	VIF
Models	EDI	Adjustments	B (95% CI)	Standard error					B (95% CI)	Standard error				
AFB1	EDI	Unadjusted	−0.063 (−0.102; −0.024)	0.02	−0.15	−3.18	0α.002	1	−0.097 (−0.14; −0.054)	0.022	−0.208	−4.454	<0.001	1
		Adjusted*	−0.07 (−0.107; −0.032)	0.019	−0.165	−3.65	<0.001	1.039	–					
AFM1	EDI	Unadjusted	–	−2.661 (−3.544; −1.778)	0.449	−0.272	−5.921	<0.001	−2.661 (−3.544; −1.778)	0.449	−0.272	−5.921	<0.001	1
		Adjusted*	–						–					
OTA	EDI	Unadjusted	−0.073 (−0.111; −0.035)	0.019	−0.176	−3.76	<0.001	1	−0.133 (−0.174; −0.092)	0.021	−0.29	−6.355	<0.001	1
		Adjusted*	−0.09 (−0.129; −0.052)	0.02	−0.218	−4.619	<0.001	1.139	–					
DON	EDI	Unadjusted	−0.0005 (−0.001; −0.00004)	0.0002	−0.102	−2.157	0.032	1	−0.002 (−0.002; −0.001)	0.0002	−0.307	−6.756	<0.001	1
		Adjusted*	−0.001 (−0.001; −0.0003)	0.0002	−0.165	−3.373	0.001	1.2	–					
T-2	EDI	Unadjusted	−0.417 (−0.815; −0.019)	0.203	−0.098	−2.057	0.04	1	−0.478 (−0.92; −0.036)	0.225	−0.101	−2.126	0.034	1
		Adjusted*	–						–					
HT-2	EDI	Unadjusted	−0.098 (−0.192; −0.004)	0.048	−0.098	−2.057	0.04	1	−0.113 (−0.217; −0.008)	0.053	−0.101	−2.126	0.034	1
		Adjusted*	–											
ZEA	EDI	Unadjusted	–	−0.125 (−0.237; −0.014)	0.057	−0.105	−2.212	0.027	−0.125 (−0.237; -0.014)	0.057	−0.105	−2.212	0.027	1
		Adjusted*	–						–					
FUM	EDI	Unadjusted	–	−0.002 (−0.005; −0.00027)	0.001	−0.105	−2.212	0.027	-0.002 (-0.005; -0.00027)	0.001	−0.105	−2.212	0.027	1
		Adjusted*	–						–					

*Adjusted for gender, age category, residence, adolescents’ education, parents’ education, and monthly income.

#### 3.7.1 AFB1

After adjusting for the variables, AFB1 EDI remained significantly associated with lower HAZ (*β* = −0.07, *p* = 0.001), indicating a significant link to stunting. AFB1 EDI was also negatively associated with BAZ (*β* = −0.097, *p* = 0.000), suggesting a significant link to thinness.

#### 3.7.2 AFM1

AFM1 EDI was negatively associated with BAZ (*β* = −2.661, *p* = 0.000), indicating a significant link to thinness.

#### 3.7.3 Ota

In the adjusted models, OTA EDI remained significantly associated with lower HAZ (*β* = −0.09, *p* = 0.000). Similarly, OTA EDI was negatively associated with BAZ (*β* = −0.133, *p* = 0.000). Hence, OTA exposure is linked to both stunting and thinness.

#### 3.7.4 Don

DON EDI was associated with lower HAZ (*β* = −0.001, *p* = 0.001) in the adjusted model and with lower BAZ (*β* = −0.002, *p* = 0.000) in the unadjusted model.

#### 3.7.5 T-2 and HT-2

After adjusting for confounders, T-2 and HT-2 EDI were no longer significant predictors of HAZ, suggesting that T-2 and HT-2 have no direct effect on HAZ. In the unadjusted models of BAZ, T-2 EDI (*β* = −0.478, *p* = 0.034) and HT-2 EDI (*β* = −0.113, *p* = 0.034) were negatively linked to BAZ. Notably, T-2 and HT-2 models showed no significant differences, likely because both were analyzed only in herbs.

#### 3.7.6 ZEA and FUM

ZEA EDI (*β* = −0.125, *p* = 0.027) and FUM EDI (*β* = −0.002, *p* = 0.027) were associated with lower BAZ scores in their unadjusted models. The ZEA and FUM models also showed no significant differences, likely because both were only analyzed in breakfast cereals.

## 4 Discussion

### 4.1 Adolescents’ dietary exposure to multi-mycotoxins and risk characterization

This study assessed Lebanese adolescents’ (10–19 years) exposure to multiple mycotoxins and the related health risks, including the association of this exposure with stunting and thinness. First, it revealed that across all food groups, adolescents mostly consumed cereals and cereal products (317.56 ± 158.12 g/day). Notably, this group was identified as the primary contributor to exposure for nearly all mycotoxins analyzed. These findings highlight the significant role that cereals and cereal products play in dietary mycotoxin exposure among adolescents, aligning with Soubra et al. (34), who stated that the Lebanese diet heavily relies on cereals, which are highly susceptible to mycotoxin contamination, such as AFT, OTA, and DON. Furthermore, AFM1 in milk and dairy products and AFB1 in thyme and thyme mixes exceeded their corresponding maximum limits, with AFM1 averaging 0.09 μg/kg, exceeding the EC limit of 0.05 μg/kg, and AFB1 averaging 11.63 μg/kg, far above the LIBNOR limit of 2 μg/kg. Thus, control measures should be implemented to prevent such high levels of contamination. Nevertheless, mycotoxins in other food groups did not exceed their respective EC maximum limits. Moreover, total AFB1 exposure was 3.41 ng/kg bw/day, which was associated with an additional 0.28 liver cancer cases/100,000 persons/year. For AFM1, total exposure was lower at 0.18 ng/kg bw/day, leading to 0.0015 liver cancer cases/100,000 persons/year. OTA exposure totaled 4.94 ng/kg bw/day, whereas total weekly OTA exposure remained below the PTWI of 100 ng/kg bw, indicating no associated significant risk of kidney diseases. DON exposure was notably high at 522.11 ng/kg bw/day. T-2 and HT-2 exposures were 0.14 ng/kg bw/day and 0.60 ng/kg bw/day, respectively. Finally, ZEA and FUM exposures were 0.34 ng/kg bw/day and 17.45 ng/kg bw/day, respectively. All total MOE values of mycotoxins analyzed, except for T-2, were below 10,000, indicating a significant risk. *To avoid overestimating health risks from non-quantifiable data, a lower-bound approach (ND = 0) was adopted for left-censored data; however, it is acknowledged that applying a middle-bound scenario (ND = LOD/2) would yield higher exposure estimates, particularly for high-consumption staple foods like bread.* All HQ values were below 1. This discrepancy between MOE and HQ values may be explained by differences in calculations with respect to the reference dose used. The MOE benchmark dose is more conservative because it does not account for uncertainty factors, unlike the reference dose of HQ, which does (50, 51). This may result in lower MOE values, which indicate a potential risk, even if HQ values are considered safe (below 1). Age group analysis indicated that early adolescents had higher estimated daily intakes of mycotoxins than late adolescents. However, no significant differences in consumption levels were reported for nearly all contaminated food groups, particularly for cereals and cereal products groups, which were the primary contributors to multiple mycotoxin exposure. Hence, this difference in exposure likely reflects the lower weight of early adolescents, with a mean of 42.71 kg, compared to late adolescents, with a mean of 60.64 kg, given that weight is a factor in the EDI calculations. Compared to national studies, average AFB1 exposure in early adolescents was 3.41 ng/kg bw/day, higher than the average AFT intake of 1.48 ng/kg bw/day reported by Soubra et al. (34) among children aged 8–13 years. Similarly, AFB1 exposure in late adolescents was 2.34 ng/kg bw/day, higher than the average AFT intake of 1.26 ng/kg bw/day among the same age group. Compared to Lebanese adults, who had a total AFB1 exposure of 1.26 ng/kg bw/day in a study conducted by Hoteit et al. (52) and 0.63–0.66 ng/kg bw/day as reported by Raad et al. (21), adolescents had a higher total AFB1 exposure of 3.41 ng/kg bw/day. However, AFM1 exposure among adolescents was 0.18 ng/kg bw/day, similar to those reported by Hassan and Kassaify (53) of 0.14 ng/kg bw/day and Raad et al. (21) of 0.22–0.31 ng/kg bw/day; however, this is lower than those reported by Daou et al. (54) of 0.495 ng/kg bw/day and Hoteit et al. (52) of 0.39 ng/kg bw/day among adults. Total dietary OTA exposure in this study was 4.94 ng/kg bw/day, nearly twice that reported by Al Ayoubi et al. (55) of 2.78 ± 1.65 ng/kg bw among Lebanese students. Similarly, weekly OTA exposure was 34.59 ng/kg bw, twice above the weekly OTA exposure reported by Soubra et al. (34) among children (8–13 years) and teenagers (14–18 years) with values of 17.57 ng/kg bw and from 14.84 ng/kg bw. Early and late adolescents’ total DON exposure was 480.58 and 315.07 ng/kg bw/day, respectively, lower than the values 545 ng/kg bw/day in children and 409 ng/kg bw/day in teenagers, reported by researchers (34). Finally, no Lebanese studies have assessed T-2, HT-2, ZEA, and FUM among adolescents, limiting our ability to compare results. Overall, when comparing adolescents’ exposure to that of adults in Lebanon, adolescents appear to have higher exposure to all tested mycotoxins, probably due to their lower weight and different consumption patterns. Comparing to international studies, total AFB1 exposure was 3.41 ng/kg bw/day, higher than that reported in China of 0.75 ng/kg bw/day for children aged 7–17 years (56) and in France (0.001–0.39 ng/kg bw/day) for children aged 3–17 years (57), yet lower than that in Hanoi (4.5–7.7 ng/kg bw/day), Vietnam, among children aged 7–18 years (58). Total AFM1 exposure (0.18 ng/kg bw/day) was higher than that in Hungary (0.022–0.071 ng/kg bw/day) (59) and France (0.054 ng/kg bw/day) (57), but less than that in Indonesia, with values of 1.23 and 1.46 ng/kg bw/day among 6–15 and 16–25 age groups, respectively (60). Total OTA exposure (4.94 ng/kg bw/day) was far above that in France (0.23–2.82 ng/kg bw/day) (57), Japan (1.20–1.56 ng/kg bw/day) (61), and the United States (0.14–0.66 ng/kg bw/day) (62). For DON, total exposure (522.11 ng/kg bw/day) was similar to that in France (544–556 ng/kg bw/day) (57), yet higher than that in Sweden (186–195 ng/kg bw/day) (63). Furthermore, T-2 exposure (0.14 ng/kg bw/day) reported far lower values than France (4–38 ng/kg bw/day) (57) and several European countries, as reported by EFSA (2.42–17.7 ng/kg bw/day) among adolescents (64). Similarly, HT-2 exposure (0.60 ng/kg bw/day) was lower than that in France (10.5–53.1 ng/kg bw/day) (57) and Europe (10.6–46.1 ng/kg bw/day) (64). ZEA exposure among early and late adolescents (0.64 and 0.24 ng/kg bw/day) were like lower bound levels in Hanoi among children aged 7–11 years and 12–18 years (0.6 and 0.4 ng/kg bw/day), but lower than the upper bound levels (5.6 and 3.7 ng/kg bw/day) (58). Finally, FUM exposure (17.45 ng/kg bw/day) was lower than that in Spain (79.25 ng/kg bw/day) (65). In general, the varied results of these studies may be attributed to several factors. These include differences in adolescents’ food consumption patterns across countries, levels of food contamination with mycotoxins, which may be influenced by geographical factors such as climate, temperature, and humidity, as well as the control measures implemented by each country. Additionally, different methodologies applied in these studies may have contributed to variations in results, such as the use of the stochastic approach by Cano-Sancho et al. (65) to assess FUM exposure and of the urinary-based intake estimations approach by Mitropoulou et al. (63) to assess DON dietary exposure.

### 4.2 Association of mycotoxin exposure with stunting and thinness among adolescents

In a study conducted by Hoteit et al. (36) among Lebanese adolescents, a stunting and thinness prevalence of 6.7 and 4.7% were reported, respectively. Based on these findings, we decided to study the way stunting and thinness are influenced by exposure to mycotoxins, after reassessing these results. Consequently, our findings showed a prevalence of stunting and thinness of 4.07 and 2.71%, respectively. Furthermore, the HAZ score showed negative associations with exposure to OTA, AFB1, and DON, unlike T-2 and HT-2, which lost significance after adjustment for many factors. As for BAZ, it showed significant unadjusted associations with exposure to all mycotoxins; the strongest were among DON, followed by OTA and AFM1, then AFB1. When the association between HAZ or BAZ and exposure to certain individual mycotoxins was tested alone, significant associations existed, indicating a potential risk. However, when sociodemographic variables were added to the models, some associations were affected, highlighting the necessity to accurately assess them while considering other contributors to growth outcomes. Notably, the prevalences of stunting and thinness observed in this national survey (4.07 and 2.71%, respectively) are lower than those reported in recent sub-national studies of Lebanese adolescents (36). This discrepancy likely stems from the national scope of the current survey, which includes a more diverse socio-economic range than previous targeted assessments of vulnerable districts. These findings should not be interpreted as an improvement in the nutritional status of the population during the economic crisis. Rather, they highlight that the crisis’s impact may be more immediately visible in dietary quality and mycotoxin exposure, due to an increased reliance on affordable, grain-based staples, than in long-term anthropometric markers like stunting, which may take longer to shift at a national scale.

A meta-analysis on the impact of AFB1 on growth outcomes reported significant negative relationships between AFB1 exposure and each of HAZ and WAZ, and thus a higher risk of stunting and underweight (66). This aligns with our results, which show a significant negative association between AFB1 exposure and both HAZ and BAZ. Further, a study in Nairobi revealed that, after adjusting for factors such as other aflatoxin exposures, sex, and age, AFM1 was negatively associated with HAZ, not WAZ, yet the other aflatoxins showed no association with either (67). Conversely, our study showed a negative association between AFM1 exposure and BAZ, not HAZ, whereas a study in Tanzania by Magoha et al. (31) showed an association between AFM1 and both WAZ and HAZ. In a study in Burkina Faso, maternal OTA exposure was not associated with either stunting or wasting among infants at 6 months of age (68). However, our study showed a significant negative association between OTA exposure and lower HAZ and BAZ among adolescents. Notably, these studies mainly focused on assessing the impact of mycotoxin exposure on growth outcomes among infants and young children (<5 years), rather than adolescents, among whom research is limited. While the co-occurrence of multiple mycotoxins (such as AFB1, OTA, and DON) was frequently observed in the analyzed food samples, it is important to clarify that this study assessed their individual associations with adolescent growth outcomes. The reported impacts on HAZ and BAZ scores represent the independent contribution of each mycotoxin, calculated through multivariate regression models that adjusted for sociodemographic confounders. Although the concurrent presence of these toxins in the diet suggests a potential for cumulative or interactive effects on nutritional status, the current analysis was not designed to quantify mathematical synergy. Future research using interaction modeling or combined risk indices (e.g., the Hazard Index approach) is warranted to elucidate further the health implications of chronic, simultaneous exposure to multiple mycotoxin mixtures in the Lebanese context. Further research is needed to understand the role of these mycotoxins on growth outcomes among adolescents and, in turn, develop targeted strategies to mitigate this public health concern.

In this study, the population-weighted cancer potency factor for AFB1 was specifically tailored to the Lebanese context by incorporating a national HBsAg prevalence of 1.55% for adolescents (47). While the resulting potency factor (0.015 cancers/100,000/year per ng/kg bw/day) is significantly lower than the global average of 0.083, it more accurately reflects Lebanon’s status as a region of low HBV endemicity. It is important to note that the JECFA cancer potency factors were originally derived from epidemiological studies of adult populations. However, in the absence of age-specific potency models for younger cohorts, these values remain the internationally recognized gold standard for baseline public health risk assessments. By utilizing localized prevalence data, this study provides a more realistic estimation of the long-term liver cancer risk attributable to dietary aflatoxin exposure among Lebanese adolescents.

### 4.3 Strengths and limitations

The strength of this study lies in being the first to assess multiple mycotoxin exposures (AFB1, AFM1, OTA, DON, T-2, HT-2, FUM, and ZEA) within the Lebanese adolescent population. By estimating associated health risks and investigating the associations between these exposures and growth outcomes—specifically stunting and thinness—this research addresses a critical gap in the Eastern Mediterranean Region. Consequently, it provides valuable insights into the public health implications of mycotoxin exposure during a vulnerable developmental stage.

Nevertheless, several limitations should be acknowledged. First, contamination data were not available for all consumed food items, which may result in an underestimation of total exposure. Furthermore, the assessment for certain toxins was limited to specific high-risk matrices (e.g., ZEA and FUM in breakfast cereals; AFM1 in dairy). While these are primary dietary sources for this cohort, this targeted approach likely excludes secondary sources such as other maize-based products or processed foods. However, such underestimation is expected to be non-differential across the sample. While it leads to more conservative risk estimates, it is unlikely to introduce significant bias into the direction of the observed associations with nutritional status (HAZ/BAZ).

A notable limitation relates to the temporal origin of the mycotoxin contamination data. In the absence of a concurrent national food-monitoring program, mean contamination values were compiled from historical studies spanning 2004 to 2022 rather than being measured directly in the foods consumed by participants. This wide temporal range introduces uncertainty, as mycotoxin occurrence is highly variable and varies with seasonal weather, storage conditions, and processing methods. Using historical averages may not perfectly reflect the acute contamination levels present during the ongoing economic crisis. Crucially, the crisis intensified after 2019, leading to frequent power outages and a potential decline in grain storage standards. Preliminary comparisons within our dataset suggest that mycotoxin levels trended higher in post-2019 samples. Therefore, the EDI and risk characterizations presented here should be interpreted as conservative estimates; the actual dietary burden at the peak of the economic collapse may exceed that captured by the historical average.

Furthermore, estimating exposure from food contamination data and intake is inherently more inaccurate than biomonitoring methods, which should be prioritized in future research. We also acknowledge that the point estimates in Supplementary Table S3 rely on mean body weights and may not reflect the higher exposure levels and shifted risks faced by lighter, younger adolescents. Additionally, the use of a Food Frequency Questionnaire (FFQ) introduces potential recall bias, as data rely on participants’ memory.

Statistically, the cross-sectional design of this study precludes the establishment of causal relationships, allowing only for the investigation of associations. We also acknowledge that mycotoxins frequently co-occur in staple foods, creating inherent collinearity among dietary exposures. While this study established a baseline by assessing individual associations, future research should utilize multivariate models or combined exposure indices to better account for interactive and cumulative effects. In this context, the Variance Inflation Factor (VIF) values of 1.0 reported for the unadjusted models are a trivial result expected from models containing only a single predictor; they do not indicate a complete absence of collinearity across the wider dietary matrix. Finally, while sociodemographic confounders were not included in the primary BAZ models because of the lack of significant bivariate associations, we recognized that the absence of a simple bivariate association does not entirely rule out residual confounding. Furthermore, since sociodemographic covariates were not included in the BAZ models due to a lack of bivariate association (*p* > 0.10), the associations between mycotoxin exposure and thinness presented in Table 9 should be interpreted as exploratory. These findings do not provide evidence of independent effects, and we caution against using them to inform public health recommendations at this stage. Future studies should employ fully adjusted models to account for potential confounding that may not be apparent in bivariate screening.

## 5 Conclusion

To conclude, our study has assessed the occurrence of mycotoxins in food items consumed by Lebanese adolescents, revealing that AFM1 in milk and dairy products and AFB1 in thyme and thyme mixes exceeded their respective EC and LIBNOR maximum limits. This highlights the need to implement strict control measures to minimize contamination levels in these food items. It has also assessed Lebanese adolescents’ exposure to multiple mycotoxins, revealing a significant health risk, as total MOE values for all mycotoxins were below 10,000, except for T-2 MOE (> 10,000) and OTA non-neo-MOE (> 200). Furthermore, AFB1 and AFM1 exposures were associated with liver cancer cases/100,000 persons/year, suggesting an urgent need for related public health policies. Finally, exposure to mycotoxins had negative impacts on growth outcomes, with exposures to OTA, AFB1, and DON associated with lower HAZ, indicating a link to stunting, and exposures to OTA, AFB1, AFM1, and DON associated with lower BAZ, suggesting a link to thinness. Future research among adolescents is needed to better understand these relationships and mitigate their harmful health implications. It’s also important to highlight the possible multiple mycotoxin contamination, which may involve combined adverse health impacts, for which research is limited yet immensely needed.

## Data Availability

The original contributions presented in the study are included in the article/Supplementary material; further inquiries can be directed to the corresponding authors.

## Footnotes

1
https://www.who.int/teams/nutrition-and-food-safety/databases/global-environment-monitoring-system-food-contamination
