# Supplementary material for: The economic crisis in Lebanon and adolescent nutritional health: a multi-mycotoxin exposure and risk assessment of stunting and thinness
Source: Front Nutr. 2026 May 15;13:1822160. doi: 10.3389/fnut.2026.1822160 (PMC13218993; doi:10.3389/fnut.2026.1822160)
Supplement: Supplementary file 1 [file Table_1.docx]

Table S1. Sociodemographic factors associated with HAZ and BAZ Scores.

|  |  | **HAZ** | | **BAZ** | |
| --- | --- | --- | --- | --- | --- |
|  | **N** | Mean ± SD | *p*-value | Mean ± SD | *p*-value |
| **Adolescents' gender** |  |  | 0.004* |  | 0.507 |
| Female | 246 | -0.27 ± 1.13 |  | 0.34 ± 1.11 |  |
| Male | 196 | 0.05 ± 1.17 |  | 0.43 ± 1.47 |  |
| **Age category** |  |  | <0.001** |  | 0.735 |
| Early Adolescents (10-13 years old) | 172 | 0.13 ± 1.31 |  | 0.41 ± 1.39 |  |
| Late Adolescents (14-18 years old) | 270 | -0.29 ± 1.00 |  | 0.36 ± 1.21 |  |
| * Significant at *p*-value <0.05 for Independent Samples T-test; ** Significant at p-value <0.001 for Independent Samples T-test. | | | | | |
| **Residence** |  |  | 0.027* |  | 0.986 |
| Beirut and Mount Lebanon | 192 | -0.24 ± 1.17 |  | 0.40 ± 1.34 |  |
| North Lebanon and Akkar | 98 | -0.26 ± 1.06 |  | 0.34 ± 1.21 |  |
| South Lebanon and Nabatieh | 95 | 0.13 ± 1.20 |  | 0.38 ± 1.26 |  |
| Baalbek-Hermel and Bekaa | 57 | 0.03 ± 1 |  | 0.36 ± 1.26 |  |
| **Adolescents Education** |  |  | 0.003* |  | 0.463 |
| Elementary School Level | 112 | 0.05 ± 1.36 |  | 0.54 ± 1.40 |  |
| Intermediate School Level | 119 | 0.06 ± 1.13 |  | 0.33 ± 1.37 |  |
| Secondary School Level | 122 | -0.28 ± 1.16 |  | 0.34 ± 1.21 |  |
| University | 89 | -0.41 ± 0.78 |  | 0.29 ± 1.09 |  |
| **School Address** |  |  | 0.050 |  | 0.961 |
| Mount Lebanon | 172 | -0.18 ± 1.20 |  | 0.40 ± 1.37 |  |
| Beirut | 22 | -0.64 ± 0.82 |  | 0.35 ± 1.04 |  |
| South Lebanon | 56 | 0.14 ± 1.26 |  | 0.26 ± 1.28 |  |
| North Lebanon | 59 | -0.12 ± 1.04 |  | 0.28 ± 1.19 |  |
| Akkar | 39 | -0.47 ± 1.01 |  | 0.44 ± 1.26 |  |
| Nabatieh | 37 | 0.11 ± 1.15 |  | 0.59 ± 1.26 |  |
| Beqaa | 31 | -0.02 ± 1.20 |  | 0.37 ± 1.03 |  |
| Baalbek-Hermel | 26 | 0.1 ± 1 |  | 0.35 ± 1.50 |  |
| **School Type** |  |  | 0.115 |  | 0.316 |
| Not Attending School | 51 | -0.39 ± 0.74 |  | 0.14 ± 1.08 |  |
| Public School | 168 | -0.02 ± 1.17 |  | 0.46 ± 1.31 |  |
| Private School | 223 | -0.16 ± 1.21 |  | 0.38 ± 1.30 |  |
| **Parents Education** |  |  | 0.022* |  | 0.958 |
| Illiterate | 19 | -0.07 ± 1.28 |  | 0.29 ± 1.27 |  |
| Elementary School Level | 54 | -0.61 ± 0.81 |  | 0.38 ± 1.37 |  |
| Intermediate School Level | 117 | -0.03 ± 1.33 |  | 0.42 ± 1.28 |  |
| Secondary School Level | 111 | -0.13 ± 1.17 |  | 0.42 ± 1.35 |  |
| University | 141 | -0.03 ± 1.04 |  | 0.32 ± 1.21 |  |
| **Monthly Income** |  |  | <0.001** |  | 0.188 |
| None | 36 | -0.06 ± 1.01 |  | 0.76 ± 1.15 |  |
| Less than 1.5-3 million L.L. | 55 | -0.63 ± 0.80 |  | 0.31 ± 1.20 |  |
| 1.5-3 million L.L. | 129 | -0.35 ± 1.20 |  | 0.18 ± 1.39 |  |
| More than 3 million L.L. | 71 | -0.16 ± 1.05 |  | 0.29 ± 1.29 |  |
| Less than 100 USD | 39 | 0.31 ± 1.19 |  | 0.58 ± 1.23 |  |
| 100-300 USD | 59 | 0.10 ± 1.09 |  | 0.49 ± 1.19 |  |
| More than 300 USD | 53 | 0.36 ± 1.31 |  | 0.51 ± 1.26 |  |

* Significant at p-value <0.05 for One-way ANOVA test; ** Significant at p-value <0.001 for One-way ANOVA test.

Table S2. Summary of linear regression model performance.

| **Models** | | | **HAZ** | | | **BAZ** | | |
| --- | --- | --- | --- | --- | --- | --- | --- | --- |
|  |  |  | **R²** | **F** | ***p-*value** | **R²** | **F** | ***p-*value** |
| **AFB1** | **EDI** | Unadjusted | 0.022 | 10.11 | 0.002 | 0.043 | 19.841 | 0.000 |
|  |  | Adjusted* | 0.142 | 10.227 | 0.000 | - | | |
| **AFM1** | **EDI** | Unadjusted | - | | | 0.074 | 35.057 | 0.000 |
|  |  | Adjusted* |  |  |  | - | | |
| **OTA** | **EDI** | Unadjusted | 0.031 | 14.14 | 0.000 | 0.084 | 40.391 | 0.000 |
|  |  | Adjusted* | 0.147 | 12.514 | 0.000 | - | | |
| **DON** | **EDI** | Unadjusted | 0.01 | 4.651 | 0.032 | 0.094 | 45.642 | 0.000 |
|  |  | Adjusted* | 0.138 | 9.912 | 0.000 | - | | |
| **T-2** | **EDI** | Unadjusted | 0.01 | 4.23 | 0.04 | 0.01 | 4.519 | 0.034 |
|  |  | Adjusted* | 0.115 | 9.444 | 0.000 | - | | |
| **HT-2** | **EDI** | Unadjusted | 0.01 | 4.23 | 0.04 | 0.01 | 4.519 | 0.034 |
|  |  | Adjusted* | 0.115 | 9.444 | 0.000 | - | | |
| **ZEA** | **EDI** | Unadjusted | - | | | 0.011 | 4.893 | 0.027 |
|  |  | Adjusted* |  |  |  | - | | |
| **FUM** | **EDI** | Unadjusted | - | | | 0.011 | 4.893 | 0.027 |
|  |  | Adjusted* |  |  |  | - | | |

*Adjusted for gender, age category, residence, adolescents' education, parents' education and monthly income.
