# Supplementary material for: The economic crisis in Lebanon and adolescent nutritional health: a multi-mycotoxin exposure and risk assessment of stunting and thinness
Source: Front Nutr. 2026 May 15;13:1822160. doi: 10.3389/fnut.2026.1822160 (PMC13218993; doi:10.3389/fnut.2026.1822160)
Supplement: Supplementary file 2 [file Table_3.docx]

Supplementary Table 3. Dietary intake, EDI and risk assessment components across food groups based on GEMS classification.

|  |  |  | **AFB1** | | | | | | **AFM1** | | | | | **OTA** | | | | | |
| --- | --- | --- | --- | --- | --- | --- | --- | --- | --- | --- | --- | --- | --- | --- | --- | --- | --- | --- | --- |
| **Food Groups** | **Food Items** | Mean dietary intake (g/day) | Mean AFB1 (µg/kg) | EDI (ng/kg bw/day) | HQ Lower Limit | HQ Upper Limit | MOE | Liver cancer risk (cancer cases/100,000 persons) | Mean AFM1 (µg/kg) | EDI (ng/kg bw/day) | HQ | MOE | Liver cancer risk (cancer cases/100,000 persons) | Mean OTA (µg/kg) | EDI (ng/kg bw/day) | HQ | MOE neo | MOE non-neo | Weekly exposure (ng/kg bw) |
| **Cereals and cereal based products** | **Bread** | 74.93 | 0.01 | 0.01 | 0.0002 | 0.0008 | 28646.26 | 0.00015 | - | | | | | 0.62 | 0.87 | 0.05 | 16668.17 | 5437.27 | 6.09 |
|  | **Breakfast cereals** | 1.21 | 0.88 | 0.02 | 0.0002 | 0.0012 | 20169.75 | 0.0003 |  |  |  |  |  | 0.62 | 0.01 | 0.0008 | 1037766.40 | 338526.56 | 0.10 |
|  | **Rice** | 71.62 | 0.50 | 0.67 | 0.0081 | 0.0393 | 599.43 | 0.01 |  |  |  |  |  | 0.65 | 0.86 | 0.05 | 16844.34 | 5494.74 | 6.03 |
|  | **Pasta** | 30.35 | 0.005 | 0.0028 | 0.0000 | 0.0002 | 141439.08 | 0.000042 |  |  |  |  |  | 0.18 | 0.10 | 0.01 | 142421.29 | 46458.81 | 0.71 |
|  | **Wheat and Bulgur** | 15.83 | 0.78 | 0.23 | 0.0028 | 0.0135 | 1738.87 | 0.00345 |  |  |  |  |  | 1.81 | 0.53 | 0.03 | 27126.44 | 8848.83 | 3.74 |
|  | **Kaak** | 0.11 | 0.46 | 0.0010 | 0.00001 | 0.0001 | 406626.66 | 0.000015 |  |  |  |  |  | 1.33 | 0.003 | 0.0002 | 5098120.00 | 1663041.90 | 0.02 |
|  | **Corn** | 8.66 |  | | | | | |  |  |  |  |  | 0.00 | 0.00 | 0.00 | - | - | - |
|  | **Flour (Manakeesh+Pizza)** | 29.14 | 0.19 | 0.10 | 0.0013 | 0.0061 | 3876.33 | 0.0015 |  |  |  |  |  | 1.32 | 0.72 | 0.04 | 20225.90 | 6597.83 | 5.02 |
|  | **Keshek** | 0.00 | 0.83 | 0.00003 | 0.0000004 | 0.000002 | 12930120.48 | 0.00000045 |  |  |  |  |  | 1.14 | 0.00004 | 0.000002 | 341258771.93 | 111320964.91 | 0.0003 |
| **Total*** |  | **231.85** | **0.46** | **1.90** | **0.02** | **0.11** | **210.50** | 0.00193218 |  |  |  |  |  | **0.85** | **3.68** | **0.20** | **3937.57** | **1284.46** | **25.78** |
| **Nuts and oilseeds** | **Nuts** | 5.38 | 0.40 | 0.04 | 0.0005 | 0.0024 | 9968.86 | 0.0006 | - | | | | | 0.25 | 0.03 | 0.0014 | 578193.62 | 188610.75 | 0.18 |
|  | **Oilseeds** | 5.88 | 0.22 | 0.02 | 0.0003 | 0.0014 | 16587.79 | 0.0003 |  |  |  |  |  | 0.08 | 0.01 | 0.0005 | 1653595.57 | 539414.28 | 0.06 |
| **Total*** |  | **11.26** | **0.31** | **0.07** | **0.0008** | **0.0038** | **6146.67** | **0.0010** |  |  |  |  |  | **0.17** | **0.03** | **0.0019** | **418625.81** | **136558.63** | **0.24** |
| **Legumes and pulses** | **Peas** | 6.91 | - | | | | | | - | | | | | 0.01 | 0.0013 | 0.0001 | 11266955.11 | 3675358.46 | 0.01 |
|  | **Beans** | 5.46 |  |  |  |  |  |  |  |  |  |  |  | 0.01 | 0.0010 | 0.0001 | 14260613.72 | 4651910.55 | 0.01 |
|  | **Lentils** | 29.85 |  |  |  |  |  |  |  |  |  |  |  | 0.105 | 0.0584 | 0.0032 | 248286.42 | 80992.74 | 0.41 |
|  | **Chickpeas** | 13.43 |  |  |  |  |  |  |  |  |  |  |  | 0.015 | 0.0038 | 0.0002 | 3861973.29 | 1259802.32 | 0.03 |
|  | **Falafel Patty** | 3.99 |  |  |  |  |  |  |  |  |  |  |  | 0.01 | 0.0007 | 0.00004 | 19495135.51 | 6359447.65 | 0.01 |
| **Total*** |  | **59.63** |  |  |  |  |  |  |  |  |  |  |  | **0.03** | **0.03** | **0.0019** | **434946.33** | **141882.49** | **0.23** |
| **Milk and dairy products** | **Milk** | 37.14 | - | | | | | | 0.04 | 0.03 | 0.14 | 20679.70 | 0.00045 | - | | | | | |
|  | **Yogurt** | 36.23 |  |  |  |  |  |  | 0.06 | 0.04 | 0.19 | 14741.70 | 0.0006 |  |  |  |  |  |  |
|  | **Labneh** | 17.08 |  |  |  |  |  |  | 0.20 | 0.06 | 0.32 | 8953.80 | 0.00009 |  |  |  |  |  |  |
|  | **Cheese** | 20.41 |  |  |  |  |  |  | 0.05 | 0.02 | 0.09 | 31794.04 | 0.0003 |  |  |  |  |  |  |
| **Total*** |  | **110.85** |  |  |  |  |  |  | **0.09** | **0.18** | **0.89** | **3206.13** | **0.0027** |  |  |  |  |  |  |
| **Fruits and fruit products** | **Olives** | 14.78 | 0.22 | 0.06 | 0.0007 | 0.0036 | 6601.19 | 0.0009 | - | | | | | 0.08 | 0.02 | 0.0012 | 658056.59 | 214662.60 | 0.15 |
|  | **Dried fruits** | 1.60 | 0.22 | 0.01 | 0.0001 | 0.0004 | 60880.94 | 0.00015 |  |  |  |  |  | 0.08 | 0.0024 | 0.0001 | 6069069.12 | 1979772.20 | 0.02 |
| **Total*** |  | **16.38** | **0.22** | **0.07** | **0.0008** | **0.0040** | **5955.46** | **0.0010** |  |  |  |  |  | **0.08** | **0.02** | **0.0014** | **593684.60** | **193664.01** | **0.17** |
| **Herbs, spices and condiments** | **Thyme** | 3.23 | 11.63 | 0.70 | 0.01 | 0.04 | 571.95 | 0.0105 | - | | | | | 1.34 | 0.08 | 0.0045 | 179945.01 | 58699.30 | 0.56 |
|  | **Herbs (Mint, parsely)** | 1.71 | 18.19 | 0.58 | 0.01 | 0.03 | 688.52 | 0.0087 |  |  |  |  |  | 4.41 | 0.14 | 0.01 | 103036.91 | 33611.35 | 0.99 |
| **Total*** |  | **4.94** | **14.91** | **1.37** | **0.02** | **0.08** | **291.40** | **0.02** |  |  |  |  |  | **2.87** | **0.26** | **0.01** | **54819.63** | **17882.54** | **1.85** |
| **Desserts and snacks** | **Croissants** | 8.43 | - | | | | | | - | | | | | 0.51 | 0.08 | 0.0044 | 182741.46 | 59611.53 | 0.56 |
|  | **Cake** | 14.69 |  |  |  |  |  |  |  |  |  |  |  | 0.46 | 0.12 | 0.01 | 116376.92 | 37962.95 | 0.87 |
|  | **Doughnut** | 3.81 |  |  |  |  |  |  |  |  |  |  |  | 0.34 | 0.02 | 0.0013 | 599954.38 | 195709.26 | 0.17 |
|  | **Chocolate** | 29.27 |  |  |  |  |  |  |  |  |  |  |  | 0.03 | 0.01 | 0.0008 | 1063367.98 | 346877.97 | 0.10 |
| **Total*** |  | **56.21** |  |  |  |  |  |  |  |  |  |  |  | **0.33** | **0.35** | **0.02** | **41789.46** | **13632.01** | **2.43** |
| **Stimulant beverages** | **Coffee, instant coffee, tea, energy drinks** | 54.84 | - | | | | | | - | | | | | 0.51 | 0.52 | 0.03 | 27817.79 | 9074.36 | 3.65 |
| **Non-alcoholic beverages** | **Milkshake** | 0.65 | - | | | | | | 0.08 | 0.0010 | 0.0049 | 586636.15 | 0.00001 | - | | | | | |
| **Alcoholic Beverages** | **Beer** | 1.62 | - | | | | | | - | | | | | 0.28 | 0.01 | 0.0005 | 1745076.51 | 569255.99 | 0.06 |
|  | **Wine, Liquor** | 0.47 |  |  |  |  |  |  |  |  |  |  |  | 1.47 | 0.01 | 0.0007 | 1132691.53 | 369491.79 | 0.09 |
| **Total*** |  | **2.09** |  |  |  |  |  |  |  |  |  |  |  | **0.87** | **0.03** | **0.0019** | **426965.73** | **139279.17** | **0.24** |

|  |  | **DON** | | | | **T-2** | | | | **HT-2** | | | | | **ZEA** | | | **FUM** | | | |
| --- | --- | --- | --- | --- | --- | --- | --- | --- | --- | --- | --- | --- | --- | --- | --- | --- | --- | --- | --- | --- | --- |
| **Food Groups** | **Food Items** | Mean DON (µg/kg) | EDI (ng/kg bw/day) | HQ | MOE | Mean T-2 (µg/kg) | EDI (ng/kg bw/day) | HQ | MOE | Mean HT-2 (µg/kg) | EDI (ng/kg bw/day) | HQ | MOE | Mean ZEA (µg/kg) | | EDI (ng/kg bw/day) | HQ | Mean FUM (µg/kg) | EDI (ng/kg bw/day) | HQ | MOE |
| **Cereals and cereal based products** | **Bread** | 176.00 | 245.76 | 0.03 | 854.50 | 0.00 | 0.00 | 0.00 | 0.00 | 0.00 | 0.00 | 0.00 | 0.00 | - | | | | - | | | |
|  | **Breakfast cereals** | 632.35 | 14.25 | 0.0018 | 14736.19 | - | | | | - | | | | | 15.10 | 0.34 | 0.0014 | 774.1 | 17.45 | 0.02 | 5732.26 |
|  | **Rice** | - | | | |  | | | |  | | | | | - | | | - | | | |
|  | **Pasta** | 62.50 | 35.35 | 0.0044 | 5940.44 |  | | | |  | | | | |  | | |  | | | |
|  | **Wheat and Bulgur** | - | | | | 0.00 | 0.00 | 0.00 | 0.00 | 0.00 | 0.00 | 0.00 | 0.00 |  | | | |  | | | |
|  | **Kaak** | 70.00 | 0.15 | 0.00002 | 1402861.99 | - | | | | - | | | | |  | | |  | | | |
|  | **Corn** | - | | | |  | | | |  | | | | |  | | |  | | | |
|  | **Flour (Manakeesh+Pizza)** | - | | | | 0.00 | 0.00 | 0.00 | 0.00 | 0.00 | 0.00 | 0.00 | 0.00 |  | | | |  | | | |
|  | **Keshek** | - | | | | - | | | | - | | | | |  | | |  | | | |
| **Total*** |  | **235.21** | **467.28** | **0.06** | **449.41** | **0.00** | **0.00** | **0.00** | **0.00** | **0.00** | **0.00** | **0.00** | **0.00** | **15.10** | | **0.34** | **0.0014** | **774.10** | **17.45** | **0.02** | **5732.26** |
| **Nuts and oilseeds** | **Nuts** | - | | | | - | | | | - | | | | | - | | | - | | | |
|  | **Oilseeds** | 62.50 | 6.85 | 0.0009 | 30654.24 | - | | | | - | | | | | - | | | - | | | |
| **Total*** |  | **62.50** | **6.85** | **0.0009** | **30654.24** |  |  |  |  |  |  |  |  |  |  |  |  |  |  |  |  |
| **Fruits and fruit products** | **Olives** | 62.50 | 17.21 | 0.0022 | 12199.01 | - | | | | - | | | | | - | | | - | | | |
|  | **Dried fruits** | 62.50 | 1.87 | 0.0002 | 112507.98 |  |  |  |  |  |  |  |  |  |  |  |  |  |  |  |  |
| **Total*** |  | **62.50** | **19.08** | **0.0024** | **11005.68** |  |  |  |  |  |  |  |  |  |  |  |  |  |  |  |  |
| **Herbs, spices and condiments** | **Thyme** | - | | | |  | | | |  | | | | |  | | |  | | | |
|  | **Herbs (Mint, parsely)** | 0.00 | 0.00 | 0.00 | 0.00 | 4.40 | 0.14 | 0.0005 | 21128.78 | 18.70 | 0.60 | 0.0020 | 4971.48 | 0.00 | | 0.00 | 0.00 | - | | | |
| **Total*** |  | **0.00** | **0.00** | **0.00** | **0.00** | **4.40** | **0.14** | **0.0005** | **21128.78** | **18.70** | **0.60** | **0.0020** | **4971.48** | **0.00** | | **0.00** | **0.00** |  |  |  |  |
| **Desserts and snacks** | **Croissants** | 50.00 | 7.86 | 0.0010 | 26730.67 | - | | | | - | | | | | - | | | - | | | |
|  | **Cake** | 60.00 | 16.43 | 0.0021 | 12781.40 |  |  |  |  |  |  |  |  |  |  |  |  |  |  |  |  |
|  | **Doughnut** | 60.00 | 4.27 | 0.0005 | 49237.64 |  |  |  |  |  |  |  |  |  |  |  |  |  |  |  |  |
|  | **Chocolate** | - | | | |  | | | |  | | | | |  | | |  | | | |
| **Total*** |  | **56.67** | **28.45** | **0.0036** | **7381.61** |  | | | |  | | | | |  | | |  | | | |
| **Stimulant beverages** | **Coffee, instant coffee, tea, energy drinks** | - | | | | - | | | | - | | | | | - | | | - | | | |
| **Non-alcoholic beverages** | **Milkshake** | - | | | |  |  |  |  |  |  |  |  |  |  |  |  |  |  |  |  |
| **Alcoholic Beverages** | **Beer** | - | | | |  |  |  |  |  |  |  |  |  |  |  |  |  |  |  |  |
|  | **Wine, Liquor** | 52.08 | 0.45 | 0.0001 | 463030.19 | - | | | | - | | | | | - | | | - | | | |
| **Total*** |  | **52.08** | **0.45** | **0.0001** | **463030.19** |  |  |  |  |  |  |  |  |  |  |  |  |  |  |  |  |
